# A Post-GWAS Replication Study Confirming the *PTK2* Gene Associated with Milk Production Traits in Chinese Holstein

**DOI:** 10.1371/journal.pone.0083625

**Published:** 2013-12-26

**Authors:** Haifei Wang, Li Jiang, Xuan Liu, Jie Yang, Julong Wei, Jingen Xu, Qin Zhang, Jian-Feng Liu

**Affiliations:** Key Laboratory of Animal Genetics, Breeding and Reproduction, Ministry of Agriculture, National Engineering laboratory for Animal Breeding, College of Animal Science and Technology, China Agricultural University, Beijing, China; Pennsylvania State University, United States of America

## Abstract

Our initial genome-wide association study (GWAS) demonstrated that two SNPs (ARS-BFGL-NGS-33248, UA-IFASA-9288) within the protein tyrosine kinase 2 (*PTK2*) gene were significantly associated with milk production traits in Chinese Holstein dairy cattle. To further validate if the statistical evidence provided in GWAS were true-positive findings, a replication study was performed herein through genotype-phenotype associations. The two tested SNPs were found to show significant associations with milk production traits, which confirmed the associations observed in the original study. Specifically, SNPs lying in the *PTK2* gene were also detected by sequencing 14 unrelated sires in Chinese Holsteins and a total of thirty-three novel SNPs were identified. Thirteen out of these identified SNPs were genotyped and tested for association with milk production traits in an independent resource population. After Bonferroni correction for multiple testing, twelve SNPs were statistically significant for more than two milk production traits. Analyses of pairwise D’ measures of linkage disequilibrium (LD) between all SNPs were also explored. Two haplotype blocks were inferred and the association study at haplotype level revealed similar effects on milk production traits. In addition, the RNA expression analyses revealed that a non-synonymous coding SNP (g.4061098T>G) was involved in the regulation of gene expression. Thus the findings presented here provide strong evidence for associations of *PTK2* variants with dairy production traits and may be applied in Chinese Holstein breeding program.

## Introduction

With the maturing of genome sequencing and high throughput SNP genotyping technologies, genome-wide association studies (GWAS) have become a routine strategy for investigating mutations underlying complex traits. So far GWAS have been successfully employed in identifying genes involving human diseases [1]–[4], economically important traits in livestock [5]–[8] and various complex traits in other species [9], [10]. Numerous candidate loci associated with respective target traits emerged from outcomes of these GWAS studies. However, it is widely accepted that GWAS is solely the first step in the process of gene discovering [11]–[15], and findings from GWAS still require further validation for ascertaining bona-fide causal variants via genetic replication as well as functional assessment [16].

Until recently,a large number of genome-wide association studies in dairy cattle focused on identifying genomic regions or SNPs associated with milk production traits [17]–[23]. Nevertheless, merely a few reports [24], [25] concerned replicated association studies involving potential functional genes. In our initial GWAS in the Chinese Holstein population [19], in addition to some functional genes such as *DGAT1* and *GHR* reported previously [26], [27], several novel potential candidate genes, proxied by hundreds of significant SNPs within these genes or in surrounding regions, were also identified. Among these novel genes, the protein tyrosine kinase 2 (*PTK2)* gene, firstly identified by our GWAS, can be considered as a promising candidate gene for milk production traits. The *PTK2* gene is located on bovine chromosome (BTA) 14. A substantial number of quantitative trait loci (QTLs) for milk production traits have been identified on BTA14 [28]–[35]. For instance, the well-known *DGAT1* gene located at ∼0.44 Mb has been functionally confirmed as a major gene affecting milk production traits [27]. Bennewitz et al. [36] paid special attention to the QTLs on BTA14 and declared that there should exist a second conditional QTL for these traits. In our previous GWAS results [19], a large proportion of the significant SNPs (61 out of 105) were located on BTA14, 59 of which lied in the reported QTL regions.

In our initial GWAS findings, it is a remarkable fact that two significant SNPs, ARS-BFGL-NGS-33248 (*P* = 1.26 E-08, n = 1815) and UA-IFASA-9288 (*P* = 2.19 E-12, n = 1815) harbored within the regions of introns 1 and 5 of the *PTK2* gene respectively, showed powerful associations with milk fat percentage from the viewpoint of statistics. These two SNPs were located in the QTL regions for fat percentage reported in previous studies [31], [34], [35] and were also observed in association with milk production traits in Holstein-Friesian cattle [22]. In addition, findings from studies in humans suggested that PTK2 plays a prominent role in the mammary gland development and function [37]–[39]. According to the basic idea of comparative genomics as well as significant signals in our initial GWAS, we therefore assumed that the *PTK2* gene could be a functional as well as positional candidate gene for milk production traits in dairy cattle. So far the association of *PTK2* polymorphisms with milk production traits has not been reported in dairy cattle.

Motivated by searching for potential casual genetic variants associated with milk production traits, we not only conducted a replication study in an independent dairy cattle population to provide convincing statistical evidence for associations of *PTK2* variants discovered in our initial GWAS study, but also performed an association study of novel SNPs within *PTK2*. Furthermore, we performed expression analyses and identified a potentially functional SNP that influences mRNA expression of *PTK2*. The results showed that the identified variants in *PTK2* may be important genetic factors implicated in milk production ability in dairy cattle and have the capability to be used in marker-assisted breeding based on further validation.

## Materials and Methods

All protocols for collection of the tissue samples of experimental individuals and phenotypic observations were reviewed and approved by the Institutional Animal Care and Use Committee (IACUC) at China Agricultural University.

### Resource Population

A daughter design was applied in this study. A total of 638 daughters together with 14 corresponding sires were collected to construct the study population. Daughters were from 15 Holstein cattle farms in Beijing, where regular and standard performance testing (dairy herd improvement, DHI) has been implemented since 1999. Estimated breeding values (EBVs) of all individuals for five milk production traits (milk yield, MY; fat yield, FY; protein yield, PY; fat percentage, FP; protein percentage, PP) were predicted by official Dairy Data Center of China based on the genetic parameters estimated via the complete DHI (Dairy Herd Improvement) data of Chinese dairy cattle population. The EBVs were then considered as “phenotypic” observations used for the subsequent analyses.

### DNA Extraction

Genomic DNA was extracted from whole blood of the daughters by DP (318) Blood DNA Kit (Tiangen Biotech Co., China) following the manufacturer’s instructions and from semen sample of the sires using a salt-out procedure [40]. The quantity and quality of isolated DNA were measured with NanoDrop™ ND-2000c Spectrophotometer (Thermo Scientific, Inc.).

### SNP Identification

A total of 32 pairs of PCR primers (File S1) were designed with Primer Premier5.0 (Premier, Canada) based on the genomic sequence of the bovine *PTK2* gene referring to Bos_taurus_UMD_3.1 assembly (NCBI Reference Sequence: AC_000171.1) to amplify all exons and partial adjacent introns. DNA pooling strategy was used to identify potential SNPs involved in the gene. DNA samples of 14 sires were selected to construct a DNA pool with equal DNA concentration of 50 ng/µl for each individual. PCRs were performed in 25 µl volume containing 50–100 ng of genomic DNA, 10 pmol of each primer, 5 mM of dNTP mix, 2.5 µl of 10×PCR buffer, 0.625 U Taq DNA polymerase (Takara Biotechnology Co. Ltd.). The PCR reaction conditions were as follows: a pre-denaturation at 95°C for 5 min, followed by 34 cycles of 30 s at 95°C, annealing from 45°C to 60°C for 35 s, 35 s at 72°C, and a final extension at 72°C for 10 min. The PCR products were sequenced using the ABI3730×l DNA analyzer (Applied Biosystems, Foster City, CA, USA).

The details of all SNPs identified have been submitted to dbSNP (http://www.ncbi.nlm.nih.gov/SNP/) and will be publicly available (accession numbers from ss836185560 to ss836185594) in dbSNP version 140.

### Genotyping

Among the identified SNPs within the region of *PTK2*, 13 SNPs was selected according to their positions for as the candidate marks. These SNPs were further genotyped for all experimental individuals using the iPLEX MassARRAY system (Sequenom Inc.).

Additionally, the same two SNPs, ARS-BFGL-NGS-33248 and UA-IFASA-9288 from the original GWAS findings were genotyped and analyzed in another independent population with the sample size of 2,284 for the sake of replication study. The information and corresponding results of these two SNPs were illustrated in File S2.

### Bioinformatics Analysis of Bovine PTK2 Protein and mRNA Structure

The potential impact of the amino acid change on the structure or function of protein was predicted by applying the two web server tools SIFT (http://sift.jcvi.org/www/SIFT.html) and PolyPhen (http://genetics.bwh.harvard.edu/pph2/). Secondary structures of the full-length bovine PTK2 mRNA were predicted by the software RNA structure4.6 [41].

### Haplotype Construction and Linkage Disequilibrium (LD) Measure

To further explore the linkage disequilibrium extent between each pair of SNPs genotyped within the *PTK2* gene, haplotypes were reconstructed for each individual using the software fastPHASE [42], and possible missing genotypes were also imputed where necessary.

Measure of pairwise LD for all pairs of SNPs within the region of the *PTK2* gene was performed using the software Haploview [43]. Accordingly, haplotype blocks where SNPs are in high LD were also determined via Haploview based on the criterion of D’. Haplotypes within each block will be employed to test their associations with phenotypes in subsequent analyses.

### Association Analyses

In the study, association analyses based on both single locus genotypes and the haplotype block were conducted to validate the effect of *PTK2* variants on milk production traits.

For single locus analyses, we adopted the same analytical strategy as employed in our initial GWAS [19]. A linear mixed regression model was fitted as follows:
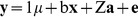
(1)where **y** is the vector of EBVs of all daughters, *μ* is the overall mean. *b* is the regression coefficient of EBVs on SNP genotypes, **x** is the vector of the SNP genotype predictors which defined as 0,1 or 2 in correspondence with the three genotypes 11,12 and 22 (assuming 1 is the minor frequency allele), **a** is the vector of residual polygenetic effects with **a∼N** (0, 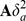) (where **A** is the additive genetic relationship matrix based on the pedigree data regarding all individuals investigated and 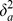 is the additive variance), and **e** is the vector of residual errors distributed as **e∼N** (0, 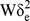), where **W** is a diagonal weight matrix with the diagonal elements calculated by 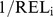 (REL*_i_* is the reliability of EBV for individual *i*) and 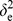 is the residual error variance. As for each SNP, the estimate of *b* and the corresponding sampling variance 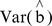 were obtained by solving the mixed model equations. Subsequently, a Wald chi-squared statistic 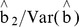 with 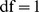 was used to determine if the SNP were associated with the dairy traits considered in this study.

For haplotype analyses considering multiple loci in high LD, we extended the original haplotype trend regression (HTR) approach [44] by allowing random effects in the regression model. For haplotypes within each haplotype block, the haplotype linear regression model with polygenic random effects was as follows:
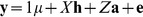
(2)where **y** is the vector of EBV of all *n* experimental daughters; *μ* is the overall mean. **1** is the n-dimensional vector with all elements equal to 1; **h** is the haplotype fixed effect vector with elements *h_i_* (*i* = 1, 2, …, *k*) being the effect of haplotype *i* of all *k* distinct haplotypes within the haplotype block; **X** is the 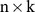 indicator matrix with the same pattern as that in [44]; **a** is the vector of the residual polygenetic effects with **a∼N** (0, 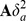) (where **A** is the additive genetic relationship matrix based on the pedigree data regarding all individuals investigated and 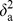 is the additive variance), and **e** is the vector of residual errors with **e∼N** (0, 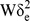) (where 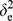 is the residual error variance and the weight matrix **W** is a diagonal matrix with each diagonal element equal to the reciprocal of the reliability of the estimate of the breeding value corresponding to individual i). For each haplotype block, the estimate of the haplotype effects vector **h** and the corresponding sampling variance 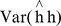 were obtained by solving the mixed model equations (MME), and a Wald chi-squared statistic 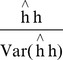 with 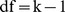 (*k* is the number of distinct haplotypes in the experimental populations within the region of haplotype block investigated) was constructed to examine whether the haplotype block was associated with the trait.

For both single locus analyses and haplotype analyses, the Bonferroni method was adopted to correct for multiple testing according to the number of SNP loci or haplotype blocks. Associations were considered significant if a raw *P* value <0.05/*N*, where *N* is the number of SNP loci or haplotype blocks tested in analyses.

The effect of a SNP on a specific trait was measured as the proportion of phenotypic variance of the trait explained by the SNP. The proportion of variance explained by a SNP was calculated as 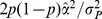, where *p* is the allele frequency of the SNP analyzed, *α* is the average effect of gene substitution calculated based on the linear mixed model employed in this study, and 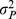 is the estimate of the phenotypic variance using the complete DHI (Dairy Herd Improvement) data of Chinese dairy cattle population.

We employed Fortran 95 to code the computing programs and they are available upon request.

### RNA Preparation, cDNA Synthesis and Quantitative Real-time PCR

To further validate the potential function of *PTK2*, we conducted differential expression analyses between different tissues as well as different genotypes at the mRNA level. Samples of eight different tissues, *i.e.*, heart, liver, lung, kidney, mammary gland, ovary, uterus and skeletal muscle, were collected from eight cows in later lactation after slaughter within 30 min. All tissue samples were frozen in liquid nitrogen and stored at −80°C. Total RNA was extracted using TRIzol Reagent (Life technologies, Carlsbad, CA, USA) following the manufacturer’s protocols. The quality of isolated RNA was checked by electrophoresis in 1% agarose gel and quantified with the NanoDropTM 2000 spectrophotometer (Thermo Scientific, USA). RNA was purified and reverse transcribed into cDNA using PrimerScript® RT reagent Kit with gDNA Eraser (TaKaRa Biotechnology Dalian Co., Ltd) according to the manufacturer’s instructions. Quantities of mRNA were then analyzed with real-time PCR using a LightCycler® 480 Real-Time PCR System (Roche, Hercules, CA, USA). The real time PCR reactions were performed in triplicate with a volume of 20 µl containing 10 µl SYBRGreen Mixture, 1 µl template of cDNA, 1 µl of each primer, 7 µl deionized water. The reaction conditions were as follows: 95°C for 5 min, 45 cycles of 95°C for 10 s, 60°C for 10 s, 72°C for 10 s. Primer sequences synthesized by Sangon Biotech (Shanghai, China) for amplifying bovine *PTK2* gene were: forward 5′-CAAGAAGAGCGTATGAGGATGG-3′, reverse 5′-GAGATGCCTGACCTGGGTAGAT-3′. The GAPDH (glyceraldehyde-3-phosphate dehydrogenase) gene was applied as an internal reference gene for normalization and the primers were: forward 5′-GGTGCTGAGTATGTGGTGGA-3′, reverse 5′-GGCATTGCTGACAATCTTGA-3′. The relative expression level was analyzed by the 2^−ΔΔCt^ method [45]. The *PTK2* gene expression levels in different tissues from three individuals were measured. To further explore the potential effects of mutations within the *PTK2* gene on its expression at mRNA level, expression levels of mammary glands from eight individuals with different genotypes at some important SNP loci were also analyzed. Gene expression data were analyzed by a t-test applying the SAS9.0 program (SAS Institute, Inc., Cary, NC, USA), with a *P* value <0.05 considered significant.

## Results

### Analyses of SNPs

**Figure 1:**
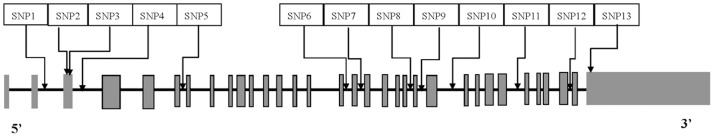
Positions of 13 genotyped SNPs in the PTK2 gene. The bars and intervals represent exons and introns respectively.

A total of thirty-three SNPs were detected in the *PTK2* gene,of which eight were located in exons and the other twenty-five in introns (Table 1). Five SNPs were synonymous substitutions and one was non-synonymous substitution (g.4061098T>G) that changed amino acid Ile into Met (NP_001068718.2:p.I981M). In accordance with the positions of these polymorphisms (Figure 1), thirteen SNPs were finally selected and genotyped for the association study. Twelve out of the selected SNPs were in Hardy-Weinberg equilibrium. The SNP12 (g.4059863A>C) did not fit with Hardy-Weinberg equilibrium (*P*<0.0001), due to the small size of the population used here. The locations and allele frequencies of the 13 SNPs are shown in Table 2.

**Table 1:** SNPs detected by sequencing in bovine PTK2 gene.

NO.	Gene region	Position on UMD_3.1	Nucleotide on UMD_3.1	Alternative nucleotide	Amino acid substitution
1	Intron2	3895208	T	G	
2	Exon2	3916050	T	C	
3	Exon2	3916093	G	A	
4	Intron3	3934715	C	T	
5	Intron6	3968566	C	T	
6	Intron6	3968605	G	A	
7	Intron8	3989212	T	C	
8	Intron10	3985663	T	C	
9	Intron12	3986703	G	A	
10	Intron12	3987085	T	C	
11	Intron13	3988746	T	C	
12	Intron15	3995419	G	A	
13	Intron15	3995746	G	C	
14	Intron16	4012367	T	C	
15	Intron17	4014815	A	G	
16	Exon21	4023970	T	C	D573D
17	Intron21	4024010	A	G	
18	Intron22	4025639	T	C	
19	Exon23	4027911	G	A	V629V
20	Intron23	4028181	C	T	
21	Intron23	4028294	C	T	
22	Intron23	4030462	C	T	
23	Intron23	4030475	G	A	
24	Intron25	4044153	C	T	
25	Intron25	4044215	T	C	
26	Intron25	4044389	C	T	
27	Intron27	4046579	C	A	
28	Exon31	4059628	C	T	L926L
29	Intron31	4059863	A	C	
30	Intron31	4059938	G	A	
31	Exon32	4061053	C	T	V996V
32	Exon32	4061098	T	G	I981M
33	Exon33	4063793	C	A	I992I

**Table 2:** Genotypes, allele frequencies and the significance of deviations from HWE.

SNP ID	Location of SNPwithin PTK2 gene	SNP position on BTA14	Allele substitution	Genotype	Genotype frequencies	Minor allele frequency	Deviations from HWE (P-value)
SNP1	Intron2	3895208	T>G	TT	0.381	0.380(G)	0.763
				TG	0.478		
				GG	0.141		
SNP2	Exon3	3916050	T>C	TT	0.975	0.013(C)	0.206
				CT	0.025		
SNP3	Exon3	3916093	G>A	GG	0.272	0.469(A)	0.406
				GA	0.516		
				AA	0.212		
SNP4	Intron3	3934715	C>T	CC	0.155	0.406(C)	0.344
				CT	0.501		
				TT	0.344		
SNP5	Intron6	3968605	G>A	GG	0.516	0.272(A)	0.108
				GA	0.423		
				AA	0.061		
SNP6	Intron16	4012367	T>C	TT	0.879	0.061(C)	0.527
				TC	0.119		
				CC	0.002		
SNP7	Intron17	4014815	A>G	AA	0.517	0.271(G)	0.088
				AG	0.423		
				GG	0.060		
SNP8	Intron21	4024010	A>G	AA	0.920	0.04(G)	0.576
				AG	0.080		
SNP9	Intron22	4025639	T>C	TT	0.516	0.271(C)	0.057
				TC	0.428		
				CC	0.056		
SNP10	Intron23	4028294	C>T	CC	0.379	0.382(T)	0.874
				CT	0.476		
				TT	0.145		
SNP11	Intron27	4046579	C>A	CC	0.366	0.388(A)	0.439
				CA	0.491		
				AA	0.143		
SNP12	Intron31	4059863	A>C	AA	0.069	0.172(A)	<0.0001
				AC	0.205		
				CC	0.726		
SNP13	Intron32	4061098	T>G	TT	0.519	0.277(G)	0.630
				TG	0.409		
				GG	0.072		

*P*-values were attained from Chi-square test of 13 SNPs. All SNP nucleotide positions were derived from the Bos_taurus_UMD_3.1 assembly (GenBank accession number: AC_000171.1).

SNP, single nucleotide polymorphism; HWE, Hardy–Weinberg equilibrium.

In addition to the above thirteen SNPs within the *PTK2* gene, the details of the two replicating SNPs (ARS-BFGL-NGS-33248, UA-IFASA-9288) are presented in Table S1 in File S2.

### Functional Prediction of the Non-synonymous SNP13

**Figure 2:**
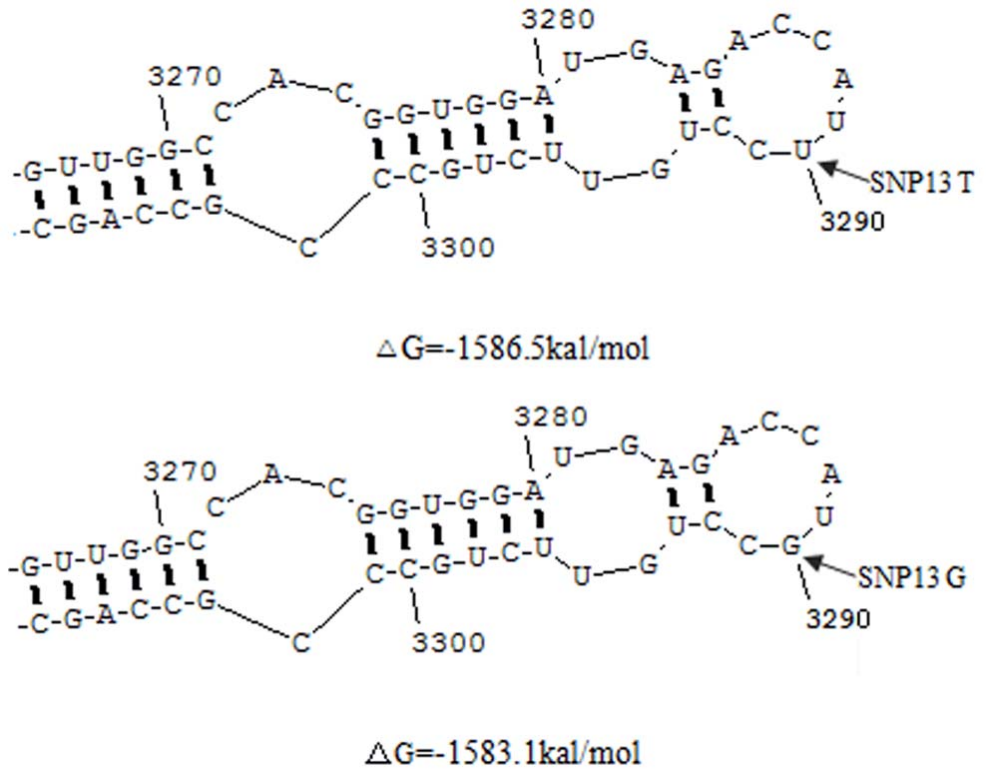
The predicted mRNA secondary structures corresponding to the PTK2 coding region alleles. Only a portion of the structure containing the non-synonymous SNP13 is displayed, while the other portion of mRNA structure shows no difference between the two alleles (indicated by arrows). The free energy (ΔG, kal/mol)) of the full-length mRNA is shown underneath the figure.

The bioinformatics analysis by applying the SIFT and PolyPhen web server tools was performed for the purpose of predicting the effect of the non-synonymous SNP13 (g.4061098T>G) on protein structure or function. The effect of this SNP was predicted to be “tolerated” and “benign” by the SIFT and PolyPhen programs respectively, suggesting that the protein structure and function may not be influenced. Afterwards, the alteration in the secondary structure of *PTK2* mRNA caused by the T/G substitution was predicted, showing little difference in mRNA structure (Figure 2). However, the free energy of *PTK2* mRNA was predicted to be changed by this substitution (−1586.5 kal/mol for T allele and −1583.1 kal/mol for G allele), indicating that the predicted mRNA structure of T allele with lower free energy might be more stable than G allele.

### Association Analyses

#### Single locus-based regression analyses

Associations between EBVs of five milk production traits and the thirteen SNPs are shown in Table 3. Of the thirteen SNPs, after Bonferroni correction for multiple testing, eight SNPs (SNP1, SNP5, SNP7, SNP8, SNP10, SNP11, SNP12, SNP13) were significantly associated with MY, PY and FP, two SNPs (SNP6, SNP9) were significant for MY, FP and PP, and one SNP (SNP3) was strongly associated with FP. However, no significant associations were observed in other SNPs (SNP2, SNP4). The significant associations between the same two SNPs from our original GWAS findings and FP were successfully confirmed (Table S2 in File S2).

**Table 3:** Associations of SNPs with EBVs of five milk production traits (LSM±SE).

SNP ID	Genotype	MY	FY	PY	FP	PP
SNP1	TT	538.94±151.12^A^	2.21±4.78	14.65±4.33^Aa^	−17.91±4.99^B^	−2.53±2.27
	TG	397.51±150.05^B^	5.06±4.75	10.79±4.29^b^	−9.39±4.95^A^	−1.88±2.25
	GG	284.31±157.94^B^	4.02±5.02	6.48±4.52^Bb^	−6.36±5.26^A^	−2.54±2.37
	P-value	0.0002*	0.1849	<.0001*	<.0001*	0.6132
	σ^2^_a_/σ^2^_P_	1.28E-02	1.46E-04	1.76E-02	1.75E-02	9.36E-05
SNP2	TT	434.81±148.54	3.87±4.69	11.62±4.26	−12.14±4.89	−2.24±2.23
	CT	508.20±200.83	4.61±6.52	16.86±5.73	−14.24±6.90	0.69±3.05
	P-value	0.5916	0.8706	0.1769	0.6700	0.1644
	σ^2^_a_/σ^2^_P_	1.14E-02	8.57E-04	1.54E-02	7.16E-03	8.01E-05
SNP3	GG	459.49±152.55	0.45±4.83	11.40±4.37	−16.83±5.05^B^	−3.25±2.29
	GA	453.63±149.91	5.43±4.74	12.37±4.29	−11.12±4.95^A^	−2.08±2.25
	AA	354.44±154.36	4.51±4.89	10.24±4.42	−8.42±5.12^A^	−1.07±2.32
	P-value	0.1532	0.0121	0.3828	0.0003*	0.0691
	σ^2^_a_/σ^2^_P_	2.70E-03	2.75E-03	4.97E-04	1.19E-02	4.43E-03
SNP4	CC	431.17±156.89	−1.32±4.99	9.82±4.49	−17.71±5.22	−3.91±2.36
	CT	424.36±149.83	4.81±4.74	11.68±4.29	−10.76±4.94	−1.84±2.25
	TT	457.63±151.76	4.69±4.81	12.55±4.35	−12.00±5.02	−2.04±2.28
	P-value	0.7771	0.0089	0.3382	0.0079	0.0890
	σ^2^_a_/σ^2^_P_	2.46E-04	4.56E-03	1.70E-03	2.97E-03	1.89E-03
SNP5	GG	344.41±150.02^C^	2.66±4.74	8.82±4.29^C^	−10.05±4.95^A^	−2.12±2.25
	GA	495.03±150.64^B^	5.12±4.77	13.62±4.32^B^	−13.12±4.97^A^	−2.19±2.26
	AA	823.33±170.87^A^	5.55±5.48	23.26±4.88^A^	−24.40±5.76^B^	−3.06±2.58
	P-value	<.0001*	0.2244	<.0001*	<.0001*	0.8023
	σ^2^_a_/σ^2^_P_	5.49E-02	6.19E-04	5.99E-02	4.59E-02	1.27E-03
SNP6	TT	462.08±148.76^A^	3.79±4.70	12.14±4.26	−13.26±4.90^B^	−2.61±2.23^B^
	TC	243.24±159.42^B^	3.91±5.08	8.36±4.56	−4.82±5.32^A^	0.77±2.39^A^
	CC	692.83±560.50^AB^	42.14±18.70	13.94±15.92	20.02±20.08^AB^	−7.34±8.62^AB^
	P-value	0.0036*	0.1070	0.1282	0.0005*	0.0032*
	σ^2^_a_/σ^2^_P_	3.38E-02	2.39E-01	6.27E-03	9.24E-02	4.75E-02
SNP7	AA	339.04±150.02^C^	2.53±4.74	8.68±4.29^C^	−9.97±4.95^A^	−2.08±2.25
	AG	501.70±150.64^B^	5.22±4.77	13.82±4.31^B^	−13.28±4.97^A^	−2.21±2.26
	GG	834.06±171.36^A^	6.04±5.49	23.36±4.89^A^	−24.24±5.78^B^	−3.30±2.59
	P-value	<.0001*	0.1558	<.0001*	<.0001*	0.6942
	σ^2^_a_/σ^2^_P_	5.73E-02	1.13E-03	6.01E-02	4.43E-02	2.05E-03
SNP8	TT	344.62±150.02^Ba^	2.66±4.74	8.82±4.29^C^	−10.05±4.95^A^	−2.12±2.25
	TC	494.59±150.61^Bb^	5.12±4.77	13.71±4.31^B^	−13.11±4.97^A^	−2.09±2.26
	CC	843.98±171.90^A^	5.54±5.51	23.05±4.91^A^	−25.15±5.79^B^	−3.92±2.59
	P-value	<.0001*	0.2246	<.0001*	<.0001*	0.4362
	σ^2^_a_/σ^2^_P_	1.58E-02	3.13E-05	1.41E-02	1.49E-02	1.79E-03
SNP9	AA	63.93±148.71	3.85±4.69	12.23±4.26	−13.26±4.90^a^	−2.58±2.23^a^
	AG	158.72±164.82	4.10±5.27	6.31±4.71	−1.44±5.53^b^	1.52±2.48^b^
	P-value	0.0001*	0.9233	0.0081	<.0001*	0.0007*
	σ^2^_a_/σ^2^_P_	5.66E-03	5.46E-03	3.32E-02	1.01E-02	2.72E-08
SNP10	CC	501.00±150.82^Aa^	1.85±4.77	13.29±4.32^Aa^	−16.89±4.98^B^	−2.70±2.26
	CT	422.36±150.25^a^	5.99±4.75	11.57±4.30^a^	−9.31±4.96^A^	−1.89±2.25
	TT	259.26±157.97^Bb^	2.35±5.02	6.45±4.52^Bb^	−7.21±5.26^A^	−1.72±2.38
	P-value	0.0012*	0.0201	0.0013*	<.0001*	0.4489
	σ^2^_a_/σ^2^_P_	1.43E-02	4.55E-03	1.50E-02	1.18E-02	6.12E-04
SNP11	CC	598.73±151.49^A^	5.93±4.79	15.94±4.34^A^	−16.12±5.01^B^	−3.16±2.27
	CA	383.22±150.00^B^	3.06±4.74	10.37±4.29^B^	−11.07±4.95^A^	−1.88±2.25
	AA	255.05±157.43^B^	2.09±5.01	6.77±4.51^B^	−7.19±5.24^A^	−1.21±2.37
	P-value	<.0001*	0.1087	<.0001*	0.0003*	0.0926
	σ^2^_a_/σ^2^_P_	2.20E-02	6.18E-04	1.97E-02	1.26E-02	2.95E-03
SNP12	AA	808.89±168.51^A^	8.45±5.39	22.09±4.82^A^	−20.81±5.67^B^	−3.73±2.54
	AC	583.80±154.65^A^	7.42±4.91	17.17±4.43^A^	−13.98±5.13^AB^	−1.49±2.32
	CC	367.38±149.03^B^	2.59±4.71	9.43±4.27^B^	−10.99±4.91^A^	−2.26±2.23
	P-value	<.0001*	0.0069	<.0001*	0.0035*	0.2912
	σ^2^_a_/σ^2^_P_	2.39E-02	8.04E-03	3.27E-02	5.38E-03	1.37E-04
SNP13	TT	521.16±150.22^A^	4.11±4.75	13.67±4.30^Aa^	−15.15±4.96^Bc^	−2.95±2.25
	TG	384.39±150.51^Ba^	3.61±4.76	10.59±4.31^a^	−10.49±4.97^b^	−1.66±2.26
	GG	157.36±167.47^Bb^	3.95±5.36	4.48±4.79^Bb^	−1.64±5.63^Aa^	−0.40±2.52
	P-value	<.0001*	0.9473	0.0002*	<.0001*	0.0521
	σ^2^_a_/σ^2^_P_	2.89E-02	7.47E-06	2.43E-02	3.36E-02	4.86E-03

A, B Within the same column with different superscripts means *P*<0.01; ^a,b^ within the same column with different superscripts means *P*<0.05.

* *P* indicates the significant association after Bonferroni correction for multiple testing at the significance level α = 0.05; σ^2^_a_/σ^2^_P_ represents the proportion of variance explained by a SNP.

#### Haplotype regression analyses

**Figure 3:**
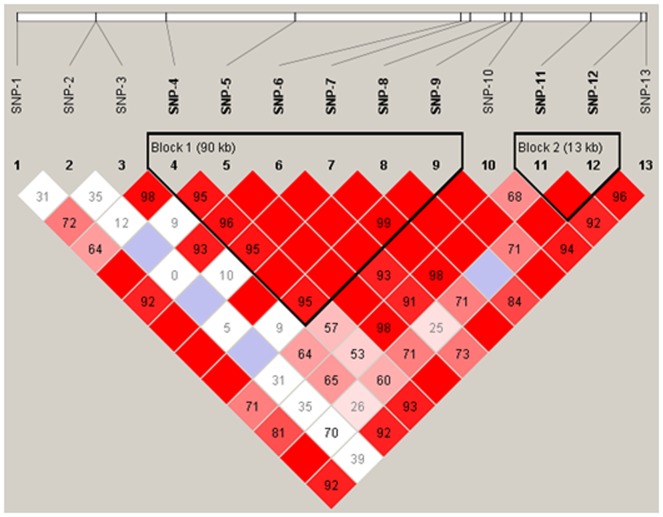
The haplotype blocks and pairwise linkage disequilibrium values (D’) for 13 SNPs in PTK2. The darker shading indicates higher linkage disequilibrium.

Two haplotype blocks were inferred (Figure 3). The block 1 consisted of 5 SNPs, which formed 4 haplotypes in the resource population. The common haplotypes TGTAAT, CGTAAT and TATGAC occurred at the frequency of 33.5%, 33.1% and 25.6% respectively. The pooled haplotypes accounted for 7.8% (Table 4). The block 2 was composed of 2 SNPs and 3 haplotypes were formed. The frequency of the haplotypes CC, AC and CA were 44.0%, 38.8% and 17.2% respectively (Table 4). The association study of the haplotypes with EBVs of five milk production traits showed that the haplotypes in block 1 were associated with MY, FP and PP and haplotypes in block 2 were correlated with MY and FP. In the analysis, the haplotype with frequency >5% was treated as a distinguishable haplotype, and those haplotypes each with relative frequency <5% were pooled into a single group.

**Table 4:** Main haplotypes of the PTK2 gene, their frequencies and associations with EBVs of five milk production traits.

Haplotypes	SNP4C>T	SNP5G>A	SNP6T>C	SNP7A>G	SNP8A>G	SNP9T>C	SNP11C>A	SNP12A>C	Frequency(%)	MY(P-value)	FP(P-value)	PP(P-value)
TGTAAT	T	G	T	A	A	T			33.5	6.94E-06*	3.26E-09*	4.01E-04*
CGTAAT	C	G	T	A	A	T			33.1			
TATGAC	T	A	T	G	A	C			25.6			
Pooled haplotypes*	C	G	C	A	G	T			7.8			
	C	G	C	A	A	T						
	C	A	T	G	A	C						
	T	A	T	A	A	C						
	T	A	T	A	A	C						
	T	A	T	G	A	T						
	T	G	C	A	A	T						
CC							C	C		1.66E-04*	7.15E-06*	8.19E-02
AC							A	C				
CA							C	A				

* : single group; *P indicates the significant association after Bonferroni correction for multiple testing at the significance level α = 0.05.

The results of the single-locus and haplotype association analyses were mostly in agreement, thus providing support to the existence of associations between these SNPs and haplotypes with milk production traits.

### Expression Analysis of the Bovine PTK2 Gene

**Figure 4:**
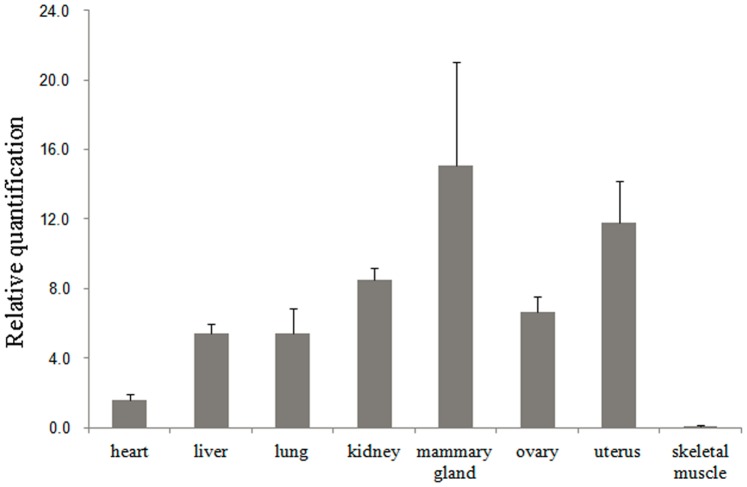
Relative quantification of the PTK2 gene in eight tissues. All relative expression levels were obtained by quantitative real-time PCR. Bars represent the mean±SE (n = 3). The values were normalized to internal GAPDH expression and the value of PTK2 in heart was randomly defined as 1.

**Figure 5:**
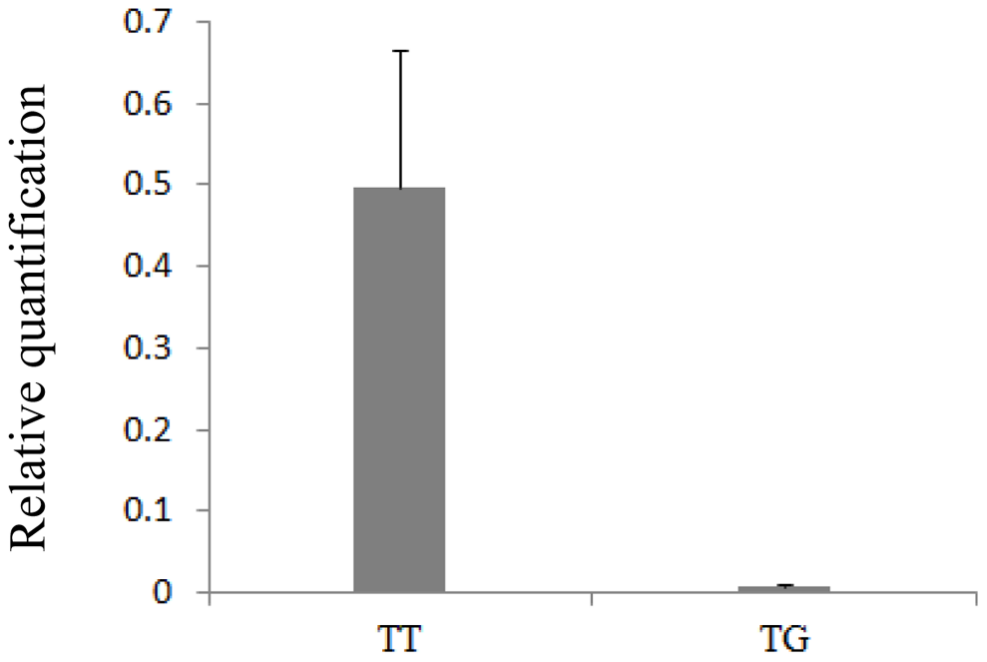
The relative quantification of PTK2 in mammary glands with different TT and TG genotypes. The internal reference gene GAPDH was used for normalization. Bars represent mean±SE (n = 4).

The relative mRNA expression of *PTK2* in different tissues was determined by quantitative real-time PCR. The results showed that bovine *PTK2* mRNA was expressed in all detected tissues, with higher expression level in mammary gland, uterus and kidney. A relatively lower expression level was found in heart and skeletal muscle tissues (Figure 4). Motivated by the results of functional prediction of the non-synonymous SNP13, the mRNA expression of *PTK2* was measured in mammary glands with different genotypes of SNP13. The results showed that mRNA expression level in mammary glands (n = 4, mean relative expression = 0.496) with the TT genotype was higher than in mammary glands (n = 4, mean relative expression = 0.008) with the TG genotype (*P* = 0.06, Figure 5), reaching more than sixty-fold. This difference did not reach the statistical significance perhaps because of the small sample size.

## Discussion

In this study, we not only replicated the associations of the same two SNPs within *PTK2* that identified in our initial GWAS study [19], but also presented a link of several novel *PTK2* variants with milk production traits. Moreover, the functional analysis revealed that the SNP13 was related to mRNA expression of the *PTK2* gene. Thus the findings presented here provide strong evidence for associations of *PTK2* variants with dairy production traits.

The EBVs of daughters herein were used as phenotypic observations in our association analyses. Although using EBVs as phenotypes may cause double-counting of relatives information, previous studies have shown that EBV-based phenotype does not significantly lower the statistical power [46], [47] compared with the other two commonly used phenotypic observations, *i.e.*, yield deviation (YD) [48] and de-regressed EBVs [49]. So far EBVs are routinely used as dependent variables in genetic association study concerning milk production traits in dairy cattle [18]–[20]. We have also compared phenotypes denoted by EBVs and de-regressed EBVs for association analyses in our initial GWAS study [19], which demonstrated that the findings based on the two different phenotypes were basically overlapped. In addition, we considered that using the same type of phenotype and analytical method employed in the initial study are very important prerequisites for exact replication [50]. Accordingly, we performed current study by applying EBVs as phenotypes to confirm the putative association from our initial GWAS study.

Two analytical methods, *i.e.*, single locus-based regression and haplotype regression analyses, were implemented to determine whether these genetic variants were associated with milk production traits. The single marker analysis was usually thought less powerful than multi-point analysis in statistics for lack of the simultaneous use of multiple marker information [51], [52]. Considering the indispensability of using the same analytical method for exact replication [50], we herein applied the same statistical method as that used in our previous GWAS study [19]. In the linear mixed model for EBVs, the polygenic random effects were treated as the random variable and the SNP genotypes as fixed effects. Under the framework of such mixed model, the biological meanings of polygenic random effects and residual errors denoted true breeding values and the sampling errors of the estimated breeding values of individuals. That is, we assumed EBVs can be explained by the SNP genotype effects investigated, the remaining polygenic effects and the residual random effects, e.g., sampling errors of EBVs. In addition, we performed multiple-point detection using haplotype regression approach to further confirm the findings from the single marker analyses. It is also notable that we succeeded in validation of significant SNPs with strong statistical signals ever after conservative Bonferroni correction for multiple testing in both single-locus and haplotype analyses, indicating the novel SNPs identified in *PTK2* could be considered as convincing genetic markers for individual selection in future cattle breeding program.

It should be mentioned that joint consideration of a set of correlated traits can offer additional information in comparison with information contained in a single trait. The previous studies concerning joint genetic linkage analyses and association analyses of multiple traits have proven that the multiple-trait analyses have statistical advantage over the single-trait analyses in detection power and evaluation of genetic effects by joint analysis of suites of correlated phenotypes [53], [54]. The reason we merely adopted the single-trait analyses herein is to keep the statistical method in our validation study consistent with that in our initial GWAS study [19], since our study goal did not focus on the search for novel genes/mutations.

The *PTK2* gene, also known as Focal Adhesion Kinase (FAK), encodes a cytoplasmic non-receptor protein tyrosine kinase which was firstly isolated from chicken embryo fibroblasts transformed by Rous sarcoma virus v-Src [55]. *PTK2* could be implicated in several signal transduction pathways such as cell motility [56], [57], microtubule stability [58], [59] and the regulation of cell-cell junctions [60]. As a major mediator of integrin signaling, *PTK2* has been found to be important for the survival, proliferation, and differentiation of mammary epithelial cells in vitro [61], [62]. In addition, *PTK2* plays a prominent role in maintaining the mammary gland development and function in vivo [63]. In this study, ubiquitous mRNA expression of *PTK2* was detected in eight different tissues, with a relatively higher expression level in the mammary gland than in other tissues, indicating its important role in the mammary gland. All these findings suggested that the participation of *PTK2* in biological processes may have vital implications for milk production in dairy cattle.

In addition to the two SNPs identified by our initial GWAS, twenty-five novel SNPs were discovered in introns. The functional role of SNPs within introns in altering gene transcriptional level has been clearly validated [64], [65]. Seven SNPs were synonymous and these SNPs always were not expected to change the function of related proteins since the non-substitution of amino acid. With better insight into the influencing factors of protein function, synonymous SNPs have increasingly received much attention. It has been reported that synonymous SNPs could impact on protein expression and thus function via altering or increasing the mRNA stability [66], [67]. In conjunction with association analyses, some associated SNPs could be selected for functional analyses in vitro to examine whether these polymorphisms are involved in the process of milk production through transcriptional alteration of the *PTK2* gene.

The SNP13 (g.4061098T>G) was a non-synonymous mutation, leading to an amino acid substitution (p.Ile981Met). Our study showed a significant association of this SNP with MY, PY and FP. Non-synonymous SNPs are always evaluated for their effect on protein stability and/or function. Some investigations have reported the impact on the transcriptional process and mRNA stability as a result of non-synonymous SNPs [68], [69]. To determine whether this SNP may modulate the expression of this gene and influence the structure or function of the related protein, real-time PCR analysis and functional prediction of non-synonymous SNP were performed. The results showed that mammary glands with the homozygous TT genotype had higher expression rates of *PTK2* mRNA than the heterozygous genotype TG. On account of genetic variation influencing gene expression and the heritability of gene expression [70], it is likely that T allele was associated with increased expression of the *PTK2* gene. Nonetheless, we cannot exclude the possibility that SNP13 was in strong linkage disequilibrium with known SNPs (SNP5, SNP7, SNP9) or undetected SNPs that disturbing gene expression. Although no predictive alteration of protein function was found, it was quite necessary to detect the PTK2 protein levels based on the genotype at SNP13 in a larger sample size and to conduct experiments on mRNA stability and functional analyses in vitro to precisely assess the effects of this polymorphism.

Based on our findings herein, a further step is needed for practical use of these variations in selection and breeding programs. Specifically, we can incorporate the information of the *PTK2* gene carried by individuals into selection program via marker-assisted selection in Chinese Holstein breeding program. The promising mutations of the *PTK2* gene can be used to increase the frequency of the marker that is positively associated with the milk production traits of interest by selecting cattles carrying two copies of the marker, and against those carrying no copies of it. As each marker in this study merely explains a small proportion of the genetic variance (Table 3), the application of marker-assisted selection may be limited. Alternatively, the marker information could be incorporated into the panel of high density SNP array in genomic selection that potentially leads to more rapid genetic gain in the dairy industry.

In conclusion, we replicated the significant associations of *PTK2* variants derived from the previous GWAS findings with milk production traits and identified a non-synonymous coding SNP (g.4061098T>G) to be involved in the regulation of gene expression. These findings strongly suggest that the associated variants are either directly responsible for the QTL effect or closely related to the causal mutation and would be useful in advanced marker-assisted selection. Further functional studies will be required to validate the effects of these markers in other populations before their applications to marker-assisted breeding in the Chinese Holstein population.

## Supporting Information

File S1 **The detailed information of primers used for PCRs of the bovine PTK2 gene.**
(DOCX)

File S2 **This file contains the description of the replication study.** **Table S1**, Information of the two SNPs used for replication study. **Table S2**, Associations of the same two SNPs identified via GWAS with EBVs of five milk production traits.
(DOCX)
