# Supplementary material for: A Post-GWAS Replication Study Confirming the PTK2 Gene Associated with Milk Production Traits in Chinese Holstein
Source: PLoS One. 2013 Dec 26;8(12):e83625. doi: 10.1371/journal.pone.0083625 (PMC3873394; doi:10.1371/journal.pone.0083625)
Supplement: File S1 — The detailed information of primers used for PCRs of the bovine PTK2 gene. (DOCX) [file pone.0083625.s001.docx]

The detailed information of primers used for PCRs of the bovine PTK2 gene.

| Amplified region | Primer | Primer sequence (5’-3’) | Product size (bp) | Annealing(°C) |
| --- | --- | --- | --- | --- |
| Exon1 | 1F | GGTCGTCGTCCGCTTTC | 465 | 57.0 |
|  | 1R | CCGGCCCTTTTGTTGTG |  |  |
| Exon2 | 2F | GAAGACAGGGACTTGATG | 403 | 52.4 |
|  | 2R | TAGAAGGGACTAAATGAC |  |  |
| Exon3 | 3F | CCCTGTGACCTGGAACAAGT | 628 | 52.0 |
|  | 3R | CACTGTCTCCATCACAAACACA |  |  |
| Exon4 | 4F | GTGGTGGGAAAGAGTGAT | 764 | 51.7 |
|  | 4R | GTCCTGTCTGGAATCGTG |  |  |
| Exon5 | 5F | GCAGTGCCTCTTCTTGTG | 526 | 50.6 |
|  | 5R | AGTACCGGCCACCTCTAT |  |  |
| Exon6 | 6F | GGGAACCTCGGGCTGTAG | 908 | 60.0 |
|  | 6R | CCGCCTGGACTCAACACC |  |  |
| Exon7 | 7F | GGGTACAGAGAACCGCTTGA | 563 | 58.4 |
|  | 7R | GGGAGCTCAGCCTTAGACTTG |  |  |
| Exon8 | 8F | TGATTGCTGCTGCCTAAT | 374 | 47.5 |
|  | 8R | TGTTCGCTAACCCAATTA |  |  |
| Exon9 | 9F | TGCCTAAACCAACTTGTCTCA | 449 | 51.6 |
|  | 9R | TTTTCTGGGAGAAAGCTCCA |  |  |
| Exon10 | 10F | TTTGGTGAATGCAGGTGGTA | 497 | 46.7 |
|  | 10R | AATCGCTGGGATGGGATTA |  |  |
| Exon11 | 11F | TTTGTGCCTGGACTGTAGC | 536 | 49.0 |
|  | 11R | CCACCCTCAAGCATTTCAT |  |  |
| Exon12 | 12F | GGACCACGGAGATTACAC | 775 | 57.6 |
|  | 12R | AGCAAACTCTGCGAGACA |  |  |
| Exon13 | 13F | TCTGGGCTCAAGTGCTTC | 452 | 48.0 |
|  | 13R | AAGGCTGTCGCTCAAATC |  |  |
| Exon14 | 14F | CTCATCTTCTTTGGTTTC | 228 | 45.0 |
|  | 14R | TTGCTTTCTGATTTCCTA |  |  |
| Exon15 | 15F | TAAGGACCTCGGGTGATA | 819 | 47.5 |
|  | 15R | AACCTGGGTCCATAGAAG |  |  |
| Exon16 | 16F | TATTATGACATGGTGGAC | 488 | 46.7 |
|  | 16R | TTATCACAGAGCAAGAAC |  |  |
| Exon17 | 17F | TTTAAGCAGTGCCGTAACAGT | 404 | 56.6 |
|  | 17R | CAATGGCAAATACCCTGAAGA |  |  |
| Exon18 | 18F | GTGTTCCTCCCACTGTTAA | 395 | 52.0 |
|  | 18R | CTCTGTACCCATGAGCCT |  |  |
| Exon19 | 19F | CCACCAGCAATTTACCAA | 548 | 59.5 |
|  | 19R | AGTGTACGTCACCCACTTT |  |  |
| Exon20 | 20F | GCTCTTCTGCTGTTGATT | 431 | 45.7 |
|  | 20R | TCCACAGTGCCACATAGA |  |  |
| Exon21 | 21F | AGCCTCTATGTGGCACTG | 785 | 52.0 |
|  | 21R | GCTCCACGGACCTGACTA |  |  |
| Exon22 | 22F | TGTTAAGTCTGTTGGCTGTG | 537 | 57.6 |
|  | 22R | GGGTCAACCGTTTACATC |  |  |
| Exon23 | 23F | CGTTCCCGTTTCTGTTTG | 748 | 46.5 |
|  | 23R | GCTGGGCTGTGAAGATTGA |  |  |
| Exon24 | 24F | TTCCTTACCTGGGCTGTG | 519 | 48.0 |
|  | 24R | CAATCATCCGTGGCTTCA |  |  |
| Exon25 | 25F | ACAAAAGGCAGATTCAC | 579 | 49.5 |
|  | 25R | TGAAGATCTCTAGGTGCT |  |  |
| Exon26 | 26F | GCCGAGTAAGGTGTTGAC | 529 | 57.6 |
|  | 26R | TCCACTGGCCTCATGTAG |  |  |
| Exon27 | 27F | AACCTGAAGCCCAAGGAA | 513 | 58.3 |
|  | 27R | TATGTCTGAGCCCGCAAT |  |  |
| Exon28 | 28F | AGCCAAGTAGGAATAAGC | 515 | 53.4 |
|  | 28R | GTTTCAGTTACGGAACGA |  |  |
| Exon29&30 | 29F | TAGGTGCAACCGTCTCGT | 842 | 53.4 |
|  | 29R | TCTGGCTCAACTCCTCTGT |  |  |
| Exon31 | 30F | CCTTCCTAGCCGCATTG | 428 | 55.0 |
|  | 30R | GTTCTCCTGCCGCTGTTC |  |  |
| Exon32 | 31F | CAGCAGTGGGTGTCCAGA | 557 | 57.0 |
|  | 31R | GACCGCTCACAGAGGAACT |  |  |
| Exon33 | 32F | CTCTGTGGGGTGGTTTCT | 833 | 55.4 |
|  | 32R | AGCCTATCCTTCATCGTTC |  |  |
