# Supplementary material for: A Post-GWAS Replication Study Confirming the PTK2 Gene Associated with Milk Production Traits in Chinese Holstein
Source: PLoS One. 2013 Dec 26;8(12):e83625. doi: 10.1371/journal.pone.0083625 (PMC3873394; doi:10.1371/journal.pone.0083625)
Supplement: File S2 — This file contains the description of the replication study. Table S1, Information of the two SNPs used for replication study. Table S2, Associations of the same two SNPs identified via GWAS with EBVs of five milk production traits. (DOCX) [file pone.0083625.s002.docx]

**Replication study**

The two significant SNPs, ARS-BFGL-NGS-33248 (P = 1.26E-08) and UA-IFASA-9288 (P = 2.19E-12) identified by our previous GWAS were used for validation in an independent Chinese Holstein population (n = 2,484) in this study. All individuals within this population were genotyped with the Illumina 54k SNP chip, which was employed for constructing a reference population used for genomic selection program in Chinese dairy cattle breeding. Accordingly, we merely selected the same two SNP markers, *i.e.*, ARS-BFGL-NGS-33248 and UA-IFASA-9288, as those identified in our initial GWAS study for replication analysis. Estimated breeding values (EBVs) of all individuals for milk production traits were treated as "phenotypic" observations. The associations of SNPs with milk production traits were analyzed by the linear mixed regression model [1] and the same statistical method as that described in the main text. The SNP with one-sided *P*-value ≤0.05 and with SNP effects in the same direction as in previous GWAS findings was treated as a successful replication.

**Table S1** Information of the two SNPs used for replication study.

| SNP ID | Location within the PTK2 gene | Position on BTA14 | Allele substitution | Minor allele frequency |
| --- | --- | --- | --- | --- |
| ARS-BFGL-NGS-33248 | Intron 1 | 3885798 | G>A | 0.216(A) |
| UA-IFASA-9288 | Intron5 | 3956956 | G>A | 0.374(A) |

**Table S2** Associations of the same two SNPs identified via GWAS with EBVs of five milk production traits (LSM±SE).

| SNP ID | Genotype | MY | FY | PY | FP | PP |
| --- | --- | --- | --- | --- | --- | --- |
| ARS-BFGL-NGS-33248 | AA | 70.9132±105.42^a^ | 4.8430±3.4024^a^ | 1.9838±3.0111 | 2.7772±3.5902^A^ | -0.2075±1.5960 |
|  | AG | 121.97±93.8410^a^ | 4.2165±2.9745^Aa^ | 4.3213±2.6878 | 0.05318±3.1072^A^ | 0.4920±1.4092 |
|  | GG | 180.96±93.2022^ab^ | 0.1308±2.9502^Bb^ | 5.5368±2.6700 | -6.8848±3.0794^B^ | -0.2788±1.3988 |
|  | P-value | 0.0099^*^ | 4.99329E-07^*^ | 0.0204^*^ | 3.20255E-17^*^ | 0.1037 |
|  | σ^2^_a_/σ^2^_P_ | 1.67E-03 | 1.03E-03 | 3.24E-03 | 6.35E-03 | 3.73E-04 |
| UA-IFASA-9288 | AA | 211.79±95.9876^A^ | 0.8210±3.0544 | 7.3525±2.7476^A^ | -7.3055±3.1977^A^ | 0.5491±1.4439 |
|  | AG | 178.78±93.5932^A^ | 2.0822±2.9651 | 6.4420±2.6809^A^ | -4.6462±3.0965^A^ | 0.7099±1.4052 |
|  | GG | 99.7209±93.6677^B^ | 1.8828±2.9676 | 3.7570±2.6830^B^ | -1.7487±3.0991^B^ | 0.5702±1.4063 |
|  | P-value | 0.0002^*^ | 0.5079 | 1.03865E-05^*^ | 5.7864E-06^*^ | 0.9153 |
|  | σ^2^_a_/σ^2^_P_ | 2.09E-03 | 3.88E-04 | 2.53E-03 | 5.02E-03 | 8.29E-06 |

^*^*P* indicates the significant association at the significance level α≤0.05; σ^2^_a_/σ^2^_P_ denotes the proportion of variance explained by a SNP.
